# Supplementary material for: Field-measured drag area is a key correlate of level cycling time trial performance
Source: PeerJ. 2015 Aug 11;3:e1144. doi: 10.7717/peerj.1144 (PMC4540006; doi:10.7717/peerj.1144)
Supplement: Appendix S1 [file peerj-03-1144-s001.docx]

**Appendix A: Methodological Details**

*Power Meter Calibration Checks*

Mechanical checks of each power meter used in this study were performed by comparing 20 units to a dynamometer from 100 to 500W (inter-unit analysis) and one unit referenced 20 times (intra-unit analysis). Results from both the inter-unit (r = 0.9998) and the intra-unit (r = 0.9999) regressions of the power meter – dynamometer relationship illustrate the reliability and validity of the units. This is similar to the findings from others (Bertucci et al., 2005). Additionally, prior to every use, each power meter was calibrated against a zero load according to the manufacturer’s instructions.

*Metabolic Cart Calibration*

Before each test, gas fractions were calibrated against a primary standard within the physiologic range (15% O_2_, 5% CO_2_), while the pneumotach was calibrated using a 3-liter syringe at 5 distinct flow rates between 50 and 300 l·min^-1^ (ATPS). In addition, a 2-minute mechanical check was performed before and after each test using the primary standard and a flow rate of 60 l·min^-1^ (ATPS). From pre- to post-mechanical checks, ventilation measures remained within 1% and gas fractions within 0.3% of original calibration values.

*Field Measured Aerodynamic and Rolling Resistance and Projected Frontal Area Profiling*

Within three weeks of the time trial, aerodynamic and rolling resistances were calculated by measuring the power versus velocity relationship while subjects rode a level section of road on the bicycle they used during the time trial, as described previously (Edwards & Byrnes, 2007; Lim et al. 2011). Briefly, since velocity affects the actual aerodynamic resistance faced by a cyclist, it is easier to describe the aerodynamic character of a cyclist independent of velocity. The constant k represents the aerodynamic character of a cyclist independent of velocity, and equals half the product of air density (ρ), projected frontal area (A_P_), and the drag coefficient (C_d_).

(1) k = 0.5 ρ · A_P_ · C_d_

With a measure of air density, it is then straightforward to remove all environmental effects and reduce equation 1 to only those terms that reflect the aerodynamic characteristics of the rider. That is, the product of the projected frontal area and the drag coefficient, a term referred to as the drag area (A_d_) (Pugh, 1974; Kyle, 1991; Grappe et al., 1997; Martin et al., 1998; Jeukendrup & Martin, 2001).

(2) A_d_ = A_P_ · C_d_

The above variables were determined in the field. A cyclist’s tractive resistance, which can be determined from field measures of power and velocity (power output · velocity^-1^ = Force (N)), was plotted against the square of velocity (v^2^) across a broad range of power outputs (100, 200, 300, and 400 Watts) and a linear regression was performed (di Prampero et al., 1979). From this relationship, the cyclist’s aerodynamic resistance (slope) and rolling resistance (intercept) were calculated. The slope of the relationship is equal to the constant k. With a value for k, along with the air density and A_P_ (described below), equation 1 can be solved for C_d_. The measured A_P_ and calculated C_d_ can then be used to determine A_d_ using equation 2.

Rolling resistance was determined as the intercept of the force versus velocity relationship described above. The following equation for rolling resistance was also used to calculate the dimensionless coefficient describing the quality of the tire and road interface (C_r_), which is dependent upon the total mass of the bicycle and rider (m_TOT_), the acceleration of gravity (g), and rolling resistance (R_r_) (Grappe et al., 1999).

(3) R_r_ = m_TOT_ · g · C_r_

*Projected Frontal Area*

Projected frontal area was determined based on the recommendations of Olds and Olive (1999). Digital photographs (Nikon Cool Pix 4300, Melville, NY) of the subjects were taken while subjects sat in a riding position on the bicycle they used during the time trial, which was mounted on a stationary trainer. The camera was set at the level of the handlebars 3 meters away from the edge of the front wheel. The shortest focal length available on the camera was used to fill the cyclist within the frame. The camera was set in its “best shot selection” mode allowing the most focused of ten automatically shot photos to be selected. Photos were taken of each cyclist in the body position used during the time trial and in different foot positions – random pedaling, with feet at the 3 and 9 o’clock position, and with feet at the 6 and 12 o’clock position. For each subject a reference board with 10 cm square tracings was also photographed. The reference board was set perpendicular to and directly over the center of the top tube of the bicycle. All photos were downloaded to an Apple G4 computer for analysis. Photographs were analyzed using NIH Image 1.62 software. This software, after calibration against a known distance or area, automatically calculates the area of a given tracing. Projected frontal area was calculated from tracings of the subject’s body and helmet only with the bicycle excluded. To enhance the objectivity of our A_P_ determinations, three independent observers performed three tracings for each subject in each body and foot position. No difference was found within or between observers for measures of projected frontal area for any subject. In addition, no difference was found within or between observers for the three distinct foot positions. The mean from all observers and all foot positions was used for data analysis and reported measures of projected frontal area.

**References:**

1. Bertucci W, Duc S, Villerius V, Permin JN, Grappe F. 2005. Validity and realiability of the PowerTap mobile cycling powermeter when compared with the SRM device. *International Journal of Sports Medicine*. 26(10): 868-73.
2. di Prampero PE, Cortili G, Mognoni P, Saibene F. 1979. Equation of motion of a cyclist. *Journal of Applied Physiology*. 47: 201-206.
3. Edwards AG, Byrnes WC. 2007. Aerodynamic characteristics as determinants of the drafting effect in cycling. *Medicine and Science in Sports and Exercise.* 39(1): 170-176.
4. Grappe F, Candau RB, Barbier B, Hoffman MD, Belli AR, Rouillon JD. 1999. Influence of tyre pressure and vertical load on coefficient of rolling resistance and simulated cycling performance. *Ergonomics.* 10: 1361-1371.
5. Grappe F, Candau RB, Belli AR, Rouillon JD. 1997. Aerodynamic drag in field cycling with special reference to the Obree's position. *Ergonomics.* 40: 1299-1311.
6. Jeukendrup AE, Martin J. 2001. Improving cycling performance: how should we spend our time and money. *Sports Medicine*. 31: 559-569.
7. Kyle CR. 1991. Wind tunnel tests of aero bicycles. *Cycling Science*. 3(3-4): 57-61.
8. Lim AC, Homestead EP, Edwards AG, Carver TC, Kram R, Byrnes WC. 2011. Measuring changes in aerodynamic/rolling resistances by cycle-mounted power meters. *Medicine and Science in Sports and Exercise*. 43(5): 853-860.
9. Martin JC, Milliken DL, Cobb JE, McFadden KL, Coggan AR. 1998. Validation of a mathematical model for road cycling power. *Journal of Applied Biomechanics*. 14: 276-291.
10. Olds T, Olive S. 1999. Methodological considerations in the determination of projected frontal area in cyclists. *Journal of Sports Science.* 17: 335-345.
11. Pugh LG. 1974. The relation of oxygen intake and speed in competition cycling and comparative observations on the bicycle ergometer. *Journal of Physiology.* 241: 795-808.
